# Assessing the role of antioxidant and pro-oxidant balance in mediating the relationship between vitamin K intake and depressive symptoms in adults

**DOI:** 10.3389/fnut.2024.1384489

**Published:** 2024-07-04

**Authors:** Lujie Wang, Shuling Huang, Zhiyi Feng, Jianyun Xiao, Gaoquan Luo, Yuan Zhang

**Affiliations:** ^1^Department of Psychiatry, Guangdong Provincial Key Laboratory of Major Obstetric Diseases, Guangdong Provincial Clinical Research Center for Obstetrics and Gynecology, The Third Affiliated Hospital of Guangzhou Medical University, Guangzhou, China; ^2^Department of Internal Medicine-Cardiovascular, Guangdong Provincial Key Laboratory of Major Obstetric Diseases, Guangdong Provincial Clinical Research Center for Obstetrics and Gynecology, The Third Affiliated Hospital of Guangzhou Medical University, Guangzhou, China; ^3^Physical examination center, Guangdong Provincial Key Laboratory of Major Obstetric Diseases, Guangdong Provincial Clinical Research Center for Obstetrics and Gynecology, The Third Affiliated Hospital of Guangzhou Medical University, Guangzhou, China; ^4^Department of Neurosurgery, Southern Theater General Hospital, Guangzhou, China; ^5^Internal Medicine, Guangdong Provincial Key Laboratory of Major Obstetric Diseases, Guangdong Provincial Clinical Research Center for Obstetrics and Gynecology, The Third Affiliated Hospital of Guangzhou Medical University, Guangzhou, China

**Keywords:** depression, vitamin K, oxidative balance scores (OBS), NHANES, mediation analysis

## Abstract

**Background:**

Growing evidence suggests a link between vitamin K (VK) intake and depression, although the underlying mechanisms remain unclear. We aimed to investigate whether oxidative balance scores (OBS) mediate the association between VK intake and depression in participants from the National Health and Nutrition Examination Survey (NHANES) 2007–2018.

**Methods:**

We analyzed data from 30,408 individuals. Dietary VK intake served as the independent variable, depression symptoms as the outcome variable, and OBS as the mediator. Multivariable logistic regression and restricted cubic splines assessed the associations. Mediation analysis was conducted to evaluate the potential mediating role of OBS.

**Results:**

Higher dietary VK intake was associated with lower depression risk in the multivariate model. Compared to the lowest log2 VK quartile, those in the higher quartiles had significantly lower depression odds (Q3: OR 0.66, 95% CI 0.55–0.78; Q4: OR 0.64, 95% CI 0.52–0.78). Additionally, a 1-unit increase in log2 VK intake was associated with a 15% decrease in depression odds (OR 0.85, 95% CI 0.81–0.90). Restricted cubic splines revealed a non-linear relationship between log2 VK and depression (p for non-linearity <0.001). Notably, OBS mediated 26.09% (*p* < 0.001) of the association between log2 VK and depression.

**Conclusion:**

Higher VK intake is associated with reduced depression risk, potentially mediated by oxidative balance. Further research is warranted to confirm causality and elucidate the underlying mechanisms.

## 1 Introduction

Current evidence indicates that depression has emerged as a significant contributor to the global burden of disease, with a lifetime risk ranging from 15–18% (1). According to the World Health Organization (WHO), depression is poised to become the predominant cause of years lived with disability worldwide by 2030 (2). Despite notable advancements in medical treatment, approximately one-third of patients do not achieve remission, despite the wide range of antidepressants available (1, 3). In recent years, there has been a growing interest in preventive approaches centered around dietary interventions, particularly involving vitamins (4, 5). In this respect, there has been an increased focus on vitamin K (VK) and its potential health benefits. A study involving female and overweight elderly Japanese individuals found an association between a deficiency in vitamin K and depressive symptoms (6). Additionally, high vitamin K supplementation has been demonstrated to significantly reduce the risk of depression in older North American individuals (7). Another study in Japan, focusing on the elderly, indicated a correlation between depression and insufficient vitamin K levels (8). A recent extensive study involving a large sample found a negative and independent correlation between vitamin K intake and the likelihood of experiencing depressive symptoms among adults in the United States (9). However, the specific mechanism through which vitamin K influences depression remains inadequately explored.

The burgeoning interest in the link between oxidative stress (OS) and depression centers upon the oxidative stress hypothesis. This hypothesis posits that an imbalance between the generation of reactive oxygen species (ROS) and the body’s antioxidant defenses can negatively impact brain structure and contribute to the development of depression (10–12). This imbalance, or level of oxidative stress, arises from a complex interplay of various factors, including dietary, lifestyle, and genetic elements. To holistically capture the combined effect of these diverse factors, the oxidative balance score (OBS) metric was developed (13). Higher OBS values generally correspond to greater antioxidant capacity within the body. Vitamin K possesses documented antioxidant and anti-inflammatory properties, shielding cells from oxidative stress (14, 15). Based on these properties, we hypothesized that vitamin K intake could influence depressive symptoms by modulating the level of oxidative stress reflected by oxidative balance scores as a marker of oxidative balance.

To validate the hypothesis that vitamin K intake is inversely associated with the risk of depressive symptoms in adults in the US, the present study utilized data from the National Health and Nutrition Examination Survey (NHANES). Furthermore, our study aimed to investigate whether oxidative balance scores mediate this association.

## 2 Materials and methods

### 2.1 Study population and ethics statement

**Figure 1:**
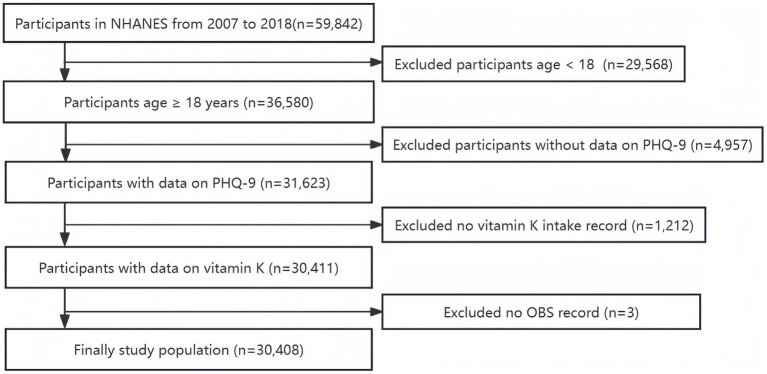
The flowchart of the patient screening process.

This study utilized data from the NHANES, an ongoing investigation into the nutrition and health status of the American population (Retrieved from https://www.cdc.gov/nchs/nhanes.htm on 15 January 2024). Before participating in the study, all individuals provided written consent. The analysis focused on information gathered from 59,842 participants during the NHANES surveys conducted from 2007 to 2018. Following the exclusion of participants under 18 years old and those lacking data on vitamin K intake, Patient Health Questionnaire-9 (PHQ-9), and observation parameters (OBS), the final cohort comprised 30,408 participants (Figure 1).

### 2.2 Evaluation of depression symptoms

In this study, we assessed depressive symptoms using PHQ-9, which aligns with the diagnostic criteria outlined in the Diagnostic and Statistical Manual of Mental Disorders-IV (DSM-IV) (16). Participants were rated on a scale ranging from 0 to 3, where 0 represented “not at all,” 1 denoted “several days,” 2 indicated “more than half the days,” and 3 reflected “nearly every day” (17). A cumulative PHQ-9 score equal to or greater than 10 was indicative of depression (18). In the present study the Cronbach’s α value was 0.839.

### 2.3 Intake of dietary vitamin K

To determine the daily dietary intake of vitamin K, we computed the average intake based on two 24-h dietary recall interviews sourced from the NHANES database (19). In instances where participants only provided data for either the first or second 24-h dietary recall, this study relied on the available one-day data. The assessment of data quality and integrity involved using the “Dietary recall status code” in NHANES, and the total VK intake data were extracted from the “Total Nutrient Intakes Files.”

### 2.4 Oxidative balance scores

The Oxidative Balance Score (OBS), adapted from Zhang et al. (20), assesses overall oxidative stress levels by analyzing sixteen dietary and four lifestyle components known to influence it (13). The dietary components are divided into two groups: dietary antioxidants and dietary pro-oxidants. The dietary antioxidants include fiber, β-carotene, riboflavin, niacin, vitamin B6, total folate, vitamin B12, vitamin C, vitamin E, calcium, magnesium, zinc, copper, and selenium. Participants are categorized into tertiles based on the distribution of these components in the study population. Those in tertile 1 to tertile 3 are assigned 0 to 2 points, respectively. Conversely, for dietary pro-oxidants such as total fat and iron, the scoring is reversed, with participants in tertile 1 receiving 2 points and those in tertile 3 scoring 0 points. The lifestyle components also consist of two groups: lifestyle antioxidants and lifestyle pro-oxidants. Physical activity is considered a lifestyle antioxidant, and participants are categorized as having low, moderate, or high physical activity levels based on the 2018 Physical Activity Guidelines for Americans (21). They are assigned 0, 1, or 2 points accordingly. Alcohol consumption is a lifestyle pro-oxidant and is scored based on sex-specific levels: consuming nonalcoholic drinks is assigned 2 points, consuming 0-15 g per day for women or 0-30 g per day for men is assigned 1 point, and consuming ≥15 g per day for women or ≥ 30 g per day for men is assigned 0 points. Smoking status is estimated using cotinine levels and assigned points ranging from 0 to 2, with higher scores indicating a higher level of smoking. Body Mass Index (BMI) is categorized as normal, overweight, or obese and assigned 2, 1, or 0 points, respectively.

### 2.5 Covariates

In this study, we identified several variables that could potentially influence the relationship between vitamin K intake, OBS, and depressive symptoms, including gender, age, race, education level, marital status, family income, diabetes, hypertension, and total energy intake. The variable representing family income in this study was expressed as the ratio of family income to poverty. “Has your doctor diagnosed you with diabetes?” was used to assess diabetes prevalence. “Has your physician diagnosed you with hypertension?” was used as to ascertain the presence/absence of hypertension.

### 2.6 Statistical analysis

Sample weights were computed based on the NHANES analysis guide. Continuous variables with normal distributions were summarized as mean ± standard deviation, while those with non-normal distributions were presented as median (interquartile range). Categorical variables were expressed as percentages. Due to the skewed distribution of dietary VK intake, the data were log2-transformed and then categorized into quartiles (Q1, Q2, Q3, and Q4). Differences between continuous variables were assessed for statistical significance using one-way analysis of variance (ANOVA) for normally distributed data or the Kruskal-Wallis test for skewed distributions. The rank-sum test evaluated differences between groups of categorical variables. A *p*-value <0.05 was statistically significant. Logistic regression models were employed to investigate the relationship between VK intake and depressive symptoms. Linear regression models explored the associations between OBS and depression, as well as VK and OBS. Three regression models were developed: Model 1 (unadjusted), Model 2 (adjusted for age, gender, race, education level, marital status, and family income), and Model 3 (adjusted for all covariables). Restricted cubic splines (RCS) were further utilized to identify potential non-linear associations between VK intake and depression.

To assess the potential mediating role of OBS in the association between log2VK and depression, we employed mediation analysis through the R package “mediation.” Five thousand bootstrap iterations, a well-established method for robust confidence interval estimation, minimized bias. We controlled for potential confounders by adjusting our analysis for gender, age, race, education level, marital status, family income, diabetes, hypertension, and total energy intake. Our analysis estimated the indirect effect size (βindirect), direct effect size (βdirect), total effect size (βtotal), proportion mediated (PM), and associated *p* values.

Data analysis was conducted using R version 4.3.1 (R Foundation for Statistical Computing, Vienna, Austria). Statistical significance was determined using a two-tailed alpha level of 0.05.

## 3 Results

### 3.1 Basic patients characteristics

Our analysis of 30,408 individuals (representing 222,496,148 US population) revealed a mean age of 46.6 years (SE = 0.26; range: 18–80) with female predominance (51.2%). The prevalence of depression was 9.08%. Compared to higher intake quartiles, participants in the lowest dietary vitamin K quartile (Q1) were younger. While the prevalence of diabetes and hypertension remained comparable across quartiles, statistically significant differences emerged in terms of gender, race, education levels, marital status, and family income (Table 1).

**Table 1:** Characteristics of 30,408 participants, National Health and Nutrition Examination Survey, the United States, 2007–2018.

Variable	Total	Q1	Q2	Q3	Q4	*p*-value
Age [mean (se), years]	46.60 (0.26)	44.35 (0.35)	45.91 (0.36)	47.44 (0.39)	48.21 (0.41)	< 0.001
Energy [mean(se), kcal]	2094.95 (8.00)	1610.02 (12.82)	2044.26 (12.21)	2268.05 (15.78)	2360.87 (16.32)	< 0.001
Gender (%)						< 0.001
Male	48.80	43.76	49.43	51.64	49.60	
Female	51.20	56.24	50.57	48.36	50.40	
Race (%)						< 0.001
Non-Hispanic White	66.38	62.09	65.93	67.17	69.42	
Non-Hispanic Black	11.27	13.91	11.09	10.19	10.36	
Other Race	7.78	6.29	6.62	7.92	9.84	
Mexican American	8.82	10.72	10.11	9.26	5.79	
Other Hispanic	5.75	6.99	6.25	5.46	4.59	
Marital status (%)						< 0.001
Married/Living with a partner	59.90	52.09	59.75	62.37	63.87	
Widowed/Divorced/Separated	18.00	21.09	17.28	17.41	16.77	
Never married	18.55	21.39	18.67	17.28	17.38	
Missing	3.56	5.44	4.30	2.94	1.99	
Family income (%)						< 0.001
IND ≤ 1.08	13.29	19.81	14.18	10.88	9.60	
1.08 < IND2.05≤1.6	16.99	21.96	17.63	16.62	12.89	
2.05 < IND ≤ 3.99≤2.8	24.24	23.30	25.32	25.82	22.58	
3.99 < IND ≤ 5≤4.9	38.29	27.10	35.02	39.82	48.55	
Missing	7.18	7.82	7.84	6.86	6.38	
Education level (%)						< 0.001
Less than 9 grade	4.58	7.83	5.13	3.65	2.41	
9-11th grade (Includes 12th grade with no diploma)	9.93	14.52	10.36	9.58	6.27	
High school graduate/GED or equivalent	22.53	26.61	25.03	22.88	16.80	
Some college or AA degree	30.90	29.37	31.56	31.78	30.72	
College graduate or above	28.50	16.16	23.68	29.17	41.80	
Missing	3.56	5.52	4.26	2.94	1.99	
Hypertension (%)						0.23
No	68.47	68.84	68.31	67.05	69.64	
Yes	31.42	31.02	31.59	32.83	30.29	
Missing	0.10	0.14	0.09	0.12	0.07	
Diabetes (%)						0.08
No	88.30	87.72	88.08	88.12	89.14	
Yes	9.61	10.31	10.07	9.74	8.55	
Borderline	2.05	1.90	1.83	2.11	2.30	
Missing	0.03	0.07	0.02	0.03	0.02	

Q1:6.64 ≤ log2VK ≤ 5.39, Q2: 5.39 < log2VK ≤ 6.15, Q3: 6.15 < log2VK ≤ 6.96, Q4: 6.96 < log2 VK ≤ 14.24.

### 3.2 Association between dietary vitamin K and depression

To assess the association between vitamin K supplementation and depression, three statistical models were implemented. Each model yielded consistent findings, albeit with varying levels of granularity. In all models, increased log2 dietary VK intake was associated with a significant decrease in depression risk. Model 1 revealed a 22% reduction (OR: 0.78, 95% CI: 0.75–0.81) per unit increase in log2 VK intake, while Models 2 and 3 demonstrated a 14% (OR: 0.86, 95% CI: 0.83–0.90) and 15% (OR: 0.85, 95% CI: 0.81–0.90) decrease, respectively. Interestingly, Model 1 and Model 3 further indicated a dose-dependent effect, with higher quartiles of VK intake associated with progressively larger reductions in depression risk: [Model 1: Q2 (25%), Q3 (47%), and Q4 (55%)], [Model 3: Q2 (12%), Q3 (34%), and Q4 (36%)]. However, Models 2 did not show a statistically significant decrease in depression for the Q2 group compared to Q1 (Table 2).

**Table 2:** Association of log2vk with depression in US adult population, NHANES 2007–2018.

Variables	Model 1 OR (95%CI) *p*-value	Model 2 OR (95%CI) *p*-value	Model 3 OR (95%CI) *p*-value
Log2vk (Continuous) (mcg) (Log 2 transform)	0.78 (0.75,0.81) < 0.001	0.86 (0.83,0.90) < 0.001	0.85 (0.81,0.90) < 0.001
Q_1_	1	1	1
Q_2_	0.75 (0.67,0.84) < 0.001	0.90 (0.80,1.01)0.08	0.88 (0.78,1.00)0.04
Q_3_	0.53 (0.46,0.61) < 0.001	0.68 (0.59,0.80) < 0.001	0.66 (0.55,0.78) < 0.001
Q_4_	0.45 (0.39,0.53) < 0.001	0.66 (0.55,0.78) < 0.001	0.64 (0.52,0.78) < 0.001
*p* for trend	<0.001	<0.001	<0.001

Outcome variables: Depression (no/yes). Exposure variables: vitamin K intake (mcg) (Log2 transform). Model 1: no covariates were adjusted. Model 2: gender, age, race, education level, marital status, and family income. Model 3: all covariates presented in Table 1 were adjusted. OR, odds ratio; CI, confidence interval.

**Figure 2:**
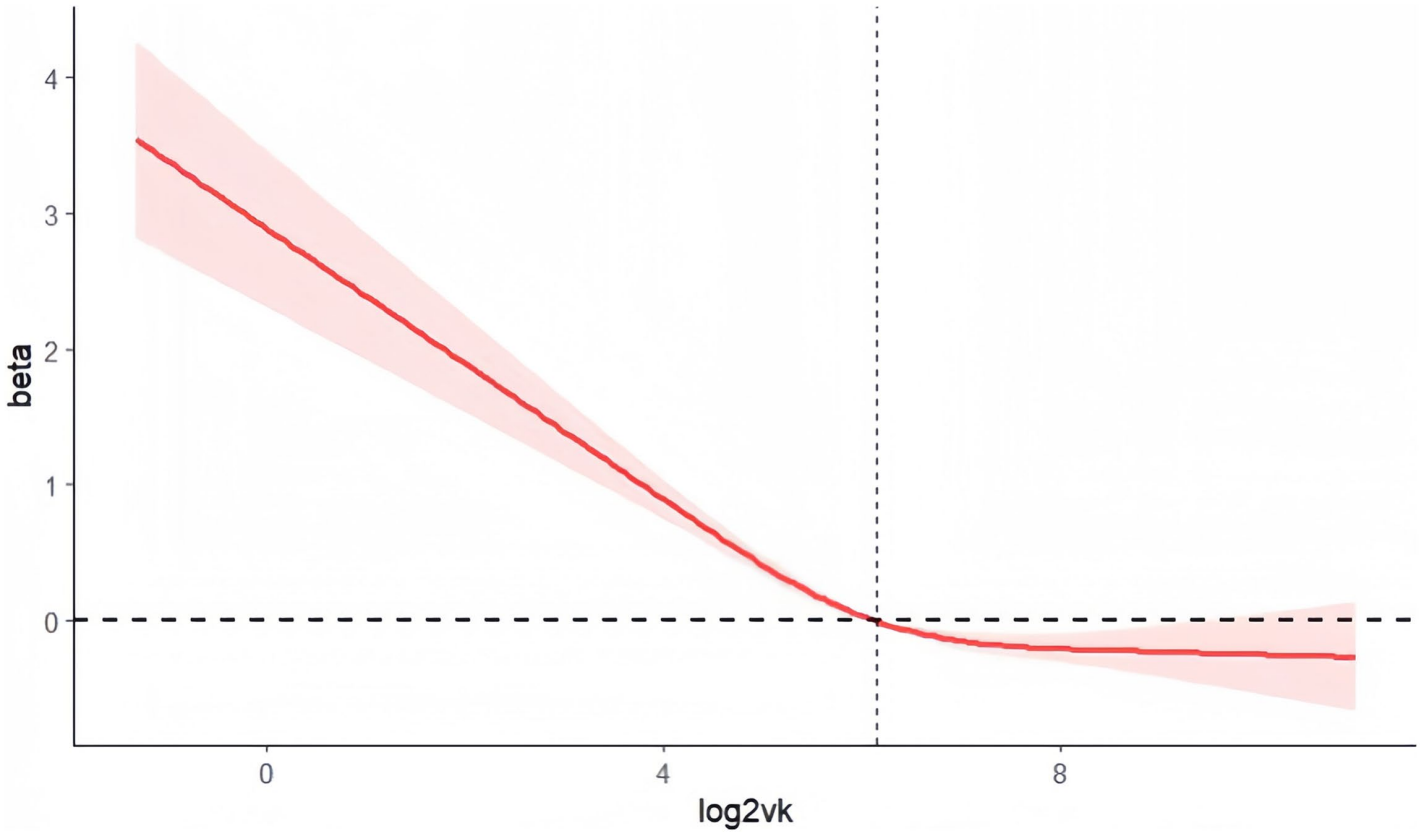
Analysis of restricted cubic spline regression. Legend: Adjusted restricted cubic spline models adjusted for Analysis of restricted cubic spline regression. Adjusted restricted cubic spline models adjusted for age, sex, race, marital status, education, poverty, diabetes, hypertension, and total energy intake. Label: P for nonlinear <0.001.

Restricted cubic splines showed a nonlinear trend (L-shape) in the association of log2VK with the risk of depressive symptoms (P for nonlinearity <0.001; Figure 2).

### 3.3 Association between OBS and depression

As shown in Table 3, linear regression analysis revealed a significant inverse association between OBS and depressive scores. Increased OBS scores were associated with lower depression scores in both the multivariable model (β coefficient − 0.04, 95% CI −0.05 to −0.03, *p* < 0.001) and individual quartile comparisons. Compared to the lowest OBS quartile (Q1), participants in Q2, Q3, and Q4 exhibited progressively lower depression scores (Q2: β −0.30, 95% CI −0.52 to −0.09, *p* = 0.01; Q3: β −0.54, 95% CI −0.77 to −0.31, *p* < 0.001; Q4: β −0.96, 95% CI −1.21 to −0.72, *p* < 0.001). These findings suggest a stronger protective effect of higher OBS against depressive symptoms.

**Table 3:** Association of OBS with depression in US adult population, NHANES 2007–2018.

Variables	Model 1 *β*(95%CI) *p-*value	Model 2 *β*(95%CI) *p-*value	Model 3 *β*(95%CI) *p-*value
OBS (Continuous)	−0.05 (−0.06, −0.04) < 0.001	−0.03 (−0.04, −0.02) < 0.001	−0.04(−0.05, −0.03) < 0.001
Q1	1	1	1
Q2	−0.47 (−0.70, −0.25) < 0.001	−0.28 (−0.49, −0.06)0.01	−0.30 (−0.52, −0.09) 0.01
Q3	−0.75 (−0.98, −0.53) < 0.001	−0.43 (−0.65, −0.21) < 0.001	−0.54 (−0.77, −0.31) < 0.001
Q4	−1.29 (−1.51, −1.07) < 0.001	−0.77 (−0.99, −0.55) < 0.001	−0.96 (−1.21, −0.72) < 0.001
*p* for trend	<0.001	<0.001	<0.001

Outcome variables: Depressive scores. Exposure variables: Oxidative Balance Scores. Model 1: no covariates were adjusted. Model 2: gender, age, race, education level, marital status, and family income. Model 3: all covariates presented in Table 1 were adjusted. β, Weighted beta estimates; CI, confidence interval.

### 3.4 Association between log2VK and OBS

Table 4 shows the associations between log2VK and OBS based on linear regressions. We found that higher levels of log2VK were associated with higher OBS.

**Table 4:** Association of log2vk with OBS in US adult population, NHANES 2007–2018.

Variables	Model 1 β (95%CI) *p*-value	Model 2 β(95%CI) *p*-value	Model 3 β(95%CI) *p*-value
log2vk (mcg) (Log 2 transform)	2.89 (2.79, 3.00) < 0.001	2.61 (2.49, 2.72) < 0.001	1.89 (1.75, 2.02) < 0.001

Outcome variables: Oxidative Balance Scores. Exposure variables: log2VK. Model 1: no covariates were adjusted. Model 2: gender, age, race, education level, marital status, and family income. Model 3: all covariates presented in Table 1 were adjusted. β, Weighted beta estimates; CI, confidence interval.

### 3.5 Mediating effect of OBS on vitamin K and depressive symptoms

**Figure 3:**
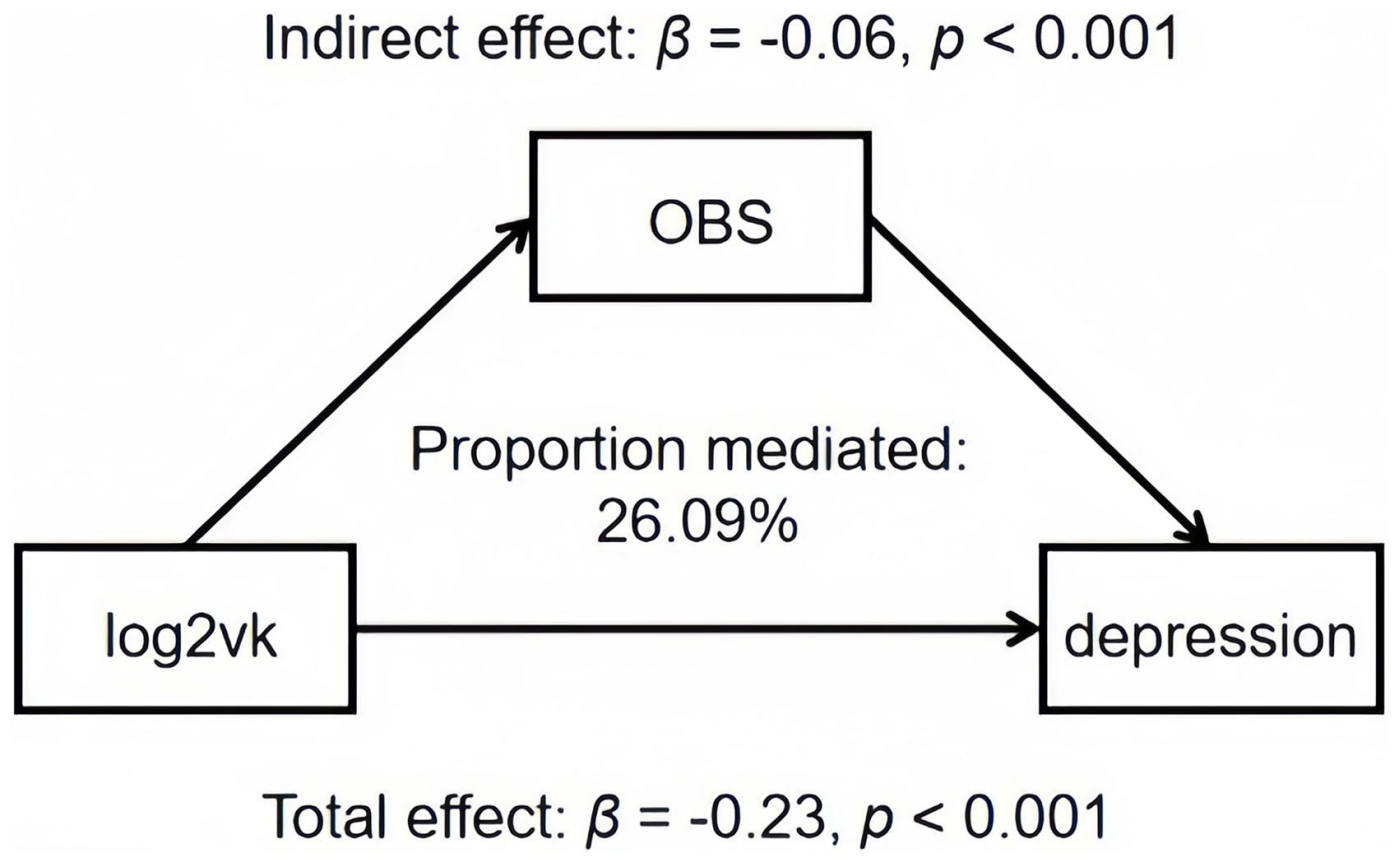
Estimated proportion of the association between log2vk and depression mediated by OBS. Models were adjusted for agender, age, race, education level, marital status, family income, diabetes, hypertension, and total energy intake. Proportion mediated (PM) = *β*indirect/*β*total. Abbreviation: OBS, oxidative balance score.

OBS was found to be a significant mediator (accounting for 26.09%) of the association between log2VK intake and depression scores, as demonstrated through mediation analyses (*p* < 0.001) (Table 5; Figure 3).

**Table 5:** Mediation analyses with OBS between log2vk and depression scores in US adult population, NHANES 2007–2018.

Mediator	Indirect effect	Direct effect	Total effect	Prop.Mediated
	βindirect (95%CI)	βdirect (95%CI)	βtotal (95%CI)	%
OBS	−0.06 (−0.07, −0.04)^***^	−0.18 (−0.24, −0.11)^***^	−0.23 (−0.29, −0.17)^***^	26.09

OBS, Oxidative Balance Scores. All analyses were adjusted for gender, age, race, education level, marital status, family income, diabetes, hypertension, and total energy intake. ^***^p < 0.001.

## 4 Discussion

The present study sheds preliminary light on the protective effect of dietary vitamin K against depression in adults aged 18–80. Despite controlling for confounding factors, the observed negative correlation between VK intake and depression displayed an L-shaped curve, indicating diminishing protective effects beyond a specific intake threshold. Moreover, this study provides the first evidence that OBS mediates the relationship between vitamin K and depression. This pioneering study opens avenues for further exploration of the interplay between VK intake, OBS, and depressive symptoms.

An increasing body of literature suggests a link between dietary vitamin K intake and depression across distinct populations. Large-scale studies have observed this negative association in adults aged 45–49 (7) and the Japanese elderly (8), with recent data further confirming a similar inverse correlation in adults in the US (9). This study contributes by extending this association to a broader age range, encompassing adults from 18 to 80 years old. However, conflicting observations exist, with one study in Spanish children reporting a positive association between vitamin K supplementation and depression (22), potentially highlighting the influence of age-specific effects and confounding factors.

There are several potential mechanisms underlying vitamin K’s antidepressant effect: Firstly, growing body of evidence highlighting elevated proinflammatory cytokine expression in both peripheral and central tissues of depression patients (23–26). Notably, the NF-kB signaling pathway plays a pivotal role in governing cytokine release (27), and interestingly, VK’s inhibitory effect on NF-kB activation has been documented (28). This suggests that VK’s anti-inflammatory properties may partially explain its protective effect against depression. Secondly, vitamin K plays a role in sphingolipid metabolism (29), with sphingolipids, particularly ceramides, recognized as markers of depression (30). Enhancing sphingolipid metabolism may ameliorate depressive symptoms (31). Research in animals indicates that inadequate vitamin K intake correlates with elevated ceramide levels in the hippocampus (32). Therefore, vitamin K may alleviate depression by enhancing the metabolism of sphingolipids, particularly ceramide. Thirdly, VK’s influence on intestinal flora could potentially regulate its antidepressant activity. VK has been shown to modulate gut microbiota (33), with emerging evidence emphasizing the crucial role of intestinal flora in modulating depressive symptoms via the brain-gut axis (34). Fourthly, VK3 has been demonstrated to inhibit monoamine oxidase (MAO) (35), a key player in depression pathogenesis (1). However, the L-shaped association observed between log2 VK and depression risk may be attributed to the synergistic effects of VK’s mitigation of NF-kB signaling and potential saturation of MAO activity. Finally, Gas-6 is a vitamin K-dependent protein extensively expressed in the nervous system, exerting anti-inflammatory effects, promoting the survival of hippocampal neurons, and regulating microglial survival (29, 36). Gas-6 induced neuronal pathways therapy holds promise for clinical significance in treating depression (37).

Numerous studies have explored the relationship between diet and depression. A recent meta-analysis has uncovered a significant inverse relationship between total dietary fiber consumption and the likelihood of depression in adults. Specifically, for every additional 5 grams of dietary fiber intake, there is a corresponding 5% decrease in the risk of developing depression (38). Another meta-analysis confirmed that both vitamin C and E supplementation are inversely associated with depression (39). Ferriani’s study of Brazilian adults identified that lower intake of vitamin B complex (B6, folate and B12) was associated with depression (40). In additional, intake of carotene and vitamin A have been proven to be negatively correlated with depression (41). Research on the relationship between dietary calcium and depression is relatively limited. However, a recent large-scale study in the American population has found a negative correlation between dietary calcium intake and depression (42). Dietary magnesium is negatively correlated with depression in a dose–response manner (43). Moreover, insufficient magnesium intake has been confirmed as a significant cause of treatment-resistant depression (44). Additionally, zinc, copper, iron and selenium have each been found to reduce the risk of depression (45, 46). The connection between a pro-oxidant lifestyle and the onset of depression has been well-established. The relationship between alcohol consumption levels and the risk of depression appears to follow a J- or U-shaped pattern. Moderate alcohol intake may alleviate depressive symptoms, whereas excessive consumption can heighten the risk of depression (47). Smoking increases the risk of depression (48), and quitting smoking can alleviate depression in individuals with or without psychiatric disorders (49). The relationship between obesity and depression is bidirectionalthe presence of one increases the risk for developing the other (50). Recent Mendelian studies have found a positive correlation and causal relationship between BMI and depression (51). On the contrary, a meta-analysis reveals that engaging in physical activity bestows substantial mental health advantages, affirming that even activity levels that fall short of public health guidelines can significantly mitigate depression (52).

Our mediation analysis revealed a compelling link between vitamin K intake and reduced depression scores, mediated by a 26.09% reduction in oxidative stress as measured by the OBS. This finding supports the hypothesis that increased vitamin K intake may alleviate depression by mitigating oxidative stress. Evidence substantiates this mechanism: Yuan et al. (53) observed *in vivo* increases in antioxidant capacity through vitamin K’s regulation of pro-oxidant and antioxidant enzymes. In another study, continuous regeneration of KH2, a potent free radical scavenger, was documented upon vitamin K supplementation *in vivo* (54). Additionally, activation of the Nrf2 antioxidant pathway, a cellular defense system against oxidative stress, has been documented following vitamin K administration (55, 56). The Nrf2 pathway is a cellular defense mechanism that helps protect cells from oxidative stress by increasing the expression of genes involved in antioxidant defense and detoxification (57). As mentioned previously, oxidative stress are important cause of depression. In recent large-scale studies, Liu et al. and Li et al. found negative correlations between OBS and depression (12, 58). Several studies have found that depressed patients and animals have significantly reduced levels of antioxidants, and increased peroxidation biomarkers (59–61). In addition, antidepressant agents can mitigate the pathogenesis of depressive disorders by upregulating the expression of antioxidant defense enzymes, further confirming the protective effect of antioxidant capacity on depression (62). This study provides valid evidence that antioxidant capacity may mediate the association between vitamin K and depression.

This study boasts several unique strengths: it pioneers the investigation of OBS mediation in the vitamin K-depression link, leverages a nationally representative sample for robust generalizability, and employs a comprehensive OBS metric capturing the influence of both dietary and lifestyle factors on individuals’ antioxidant status.

Despite the promising findings, recognizing the study’s limitations is crucial. The cross-sectional design precludes definitive conclusions about causality between dietary vitamin K and depression. Additionally, our reliance on 24-h dietary and the data collection method of OBS recalls introduces potential recall and measurement bias that may cloud the accuracy of the observed associations. Lastly, the possibility of unaddressed confounders such as pollutants, drugs, fatty acids and other diseases influencing the results cannot be dismissed. This underscores the need for further research employing robust methodologies to clarify the causal relationship and elucidate the underlying mechanisms.

## 5 Conclusion

This study demonstrates an L-shaped relationship between dietary vitamin K supplementation and depression in adults aged 18–80. Notably, OBS appears to partially mediate this association, suggesting a potential mechanism that warrants further investigation. The study suggests that dietary supplementation of VK may help reduce depressive symptoms. Additionally, maintaining an antioxidant-rich diet and behavioral patterns can contribute to the antidepressant effects of VK. To establish this link and elucidate its underlying biological pathways, rigorous longitudinal studies are imperative.

## Data availability statement

The datasets presented in this study can be found in online repositories. The names of the repository/repositories and accession number(s) can be found at: https://www.cdc.gov/nchs/nhanes.htm.

## Ethics statement

The studies involving humans were approved by National Health and Nutrition Examination Survey. The studies were conducted in accordance with the local legislation and institutional requirements. Written informed consent for participation in this study was provided by the participants' legal guardians/next of kin.

## Author contributions

LW: Conceptualization, Formal analysis, Writing – original draft, Writing – review & editing. SH: Writing – review & editing. ZF: Data curation, Resources, Writing – review & editing. JX: Data curation, Software, Writing – original draft. GL: Project administration, Supervision, Writing – review & editing. YZ: Supervision, Visualization, Writing – review & editing.
